# Advanced N-Graphene-Based Electromagnetic Shields via Microwave Plasma Induced Synthesis

**DOI:** 10.3390/nano16211358

**Published:** 2026-10-25

**Authors:** Elena Tatarova, Neli Bundaleska, Edgar Felizardo, Ana Dias, Antonio Silva, Miroslav Abrashev, Jivko Kissovski, Janez Zavašnik, Uroš Cvelbar, Nenad Bundaleski, Orlando M. N. D. Teodoro, Amelia Almeida, Milena Georgieva, Todor Karadimov, Bruno Gonçalves

**Affiliations:** 1 Instituto de Plasmas e Fusão Nuclear, Instituto Superior Técnico, Universidade de Lisboa, 1049-001 Lisboa, Portugal; 2 GTechPlasma, Instituto Superior Técnico, Av. Prof. Dr. Cavaco Silva, n.º 1, Porto Salvo, 2744-016 Lisboa, Portugal; 3 Faculty of Physics, Sofia University, 1164 Sofia, Bulgaria; 4 Department of Gaseous Electronics F6, Jožef Stefan Institute, Jamova cesta 39, SI-1000 Ljubljana, Slovenia; 5 Centro de Física e Investigação Tecnológica, Departamento de Física, Faculdade de Ciencias e Tecnologia, Universidade Nova de Lisboa, 2829-516 Caparica, Portugal; 6 Centre of Physics and Engineering of Advanced Materials, Instituto Superior Técnico, Universidade de Lisboa, 1049-001 Lisboa, Portugal

**Keywords:** N-graphene nanocomposites, plasma synthesis, EM wave shielding

## Abstract

Here, a plasma-enabled strategy is presented for the controllable fabrication of ultralight nanocomposites with absorption-dominated EM-shielding performance across the 5–70 GHz frequency range. The fabricated nanocomposites exhibit average microwave absorption exceeding 60% across the 5–25 GHz frequency range and 70% across the 25–70 GHz range while maintaining a constant thickness of 2 mm and a fixed filler content of 15 wt%. Plasma-driven nanoscale engineering and multiphase hybridization synergistically enhance dielectric and magnetic loss mechanisms, resulting in effective absorption bandwidths in the C-band (2.1 GHz), Ku-band (3.4 GHz), Ka-band (8 GHz) and V-band (4 GHz). The plasma process produces free-standing nitrogen-doped graphene sheets uniformly decorated with Fe_x_O_y_, MnO_x_, and TiO_x_ nanoparticles, which are subsequently incorporated into a polymer matrix. The microwave plasma environment simultaneously enables graphene nanosheet synthesis, metal oxide nanoparticles formation, surface functionalization, multiphase generation, and the anchoring of nanoparticles onto the graphene scaffold. Using low-cost precursors such as acetonitrile and micron-sized metal oxides, the process achieves production rates of up to 50 mg min^−1^. Overall, this one-step plasma-enabled approach provides a low-cost, scalable, and efficient route for manufacturing lightweight, high-performance EM wave absorbers.

## 1. Introduction

The widespread use of portable devices, 5G or future 6G wireless communication technologies inevitably generates a substantial amount of electromagnetic (EM) radiation at different frequencies. EM pollution causes problems such as system failures, data loss, and signal disturbance and poses a serious threat to communication security and most importantly human health [1,2]. To this end, EM functional materials play an increasingly important role in solving EM pollution in both modern civil and military fields and have a wide range of applications, including commercial and scientific electronics, antenna systems, space exploration, satellite communication, advanced driving assisted systems, millimeter-wave wireless LAN and medical devices. With the development of wearable electronics and rising stealth technology, tremendous interest has been also focused on high-efficiency radar-absorbing coatings and flexible wearable absorbers [3].

When electromagnetic waves strike the EM shield, the occurrence can be reflection, absorption, transmission, and multiple internal reflections [4]. Reflection is the main protective mechanism in a homogeneous conducting material, where the materials must possess mobile charge carriers to interact with the EM waves. Absorption depends on the thickness of the shield, and the absorption capacity increases when the material acquires electric and magnetic dipoles in contact with EM waves. Multiple internal reflections are the third shielding mechanism, which usually decreases if the shield is thinner than the skin depth. Reflection-induced secondary electromagnetic pollution is a significant concern; consequently, shielding materials are designed to achieve high EM absorption efficiency while minimizing reflected EM waves [5]. Electromagnetic attenuation is key for an ideal electromagnetic absorbing material. Excellent attenuation generally depends on two features: dielectric loss and magnetic loss [6,7].

Materials with tunable electromagnetic (EM) properties have long been a central focus in advanced materials research, particularly due to the need for precise control over dielectric and magnetic loss mechanisms governing EM absorption. Traditional materials, such as ferrites, magnetic metals, and conductive polymers, are classical materials for EM wave attenuation applications but are highly limited in practical demands due to their high density, poor environmental stability, and difficulty in processing, and metal-based materials (e.g., brass, copper, nickel, steel, etc.) suffer from corrosion and are heavy in weight [8]. Furthermore, conducting polymer composites earned recognition in comparison with metal-based composites due to their flexibility, light weight and resistance to corrosion [9]. However, polymer-based materials provide less shielding, as they are less conductive, have limited mechanical properties, and present lower temperature resistance. It is also difficult to use polymers under extreme temperatures. Recently, the boom of different kinds of nanomaterials, including zero-dimensional (0D) nanoparticles (NPs) [10], 1D carbon nanotubes (CNTs) [11], carbon nanofibers [12], carbon microspheres [13,14] and 2D nano-planes such as graphene [15], MXene [16], MoS_2_ [17], PANI@CoFe_2_O_4_ organohydroges [18], significantly promotes the advancement of EM protection technology. Toward “thin, wide, light, and strong” EM-shielding materials, 2D nanomaterials, in particular graphene, are undoubtedly the preferred choice due to tunable electrical properties and abundant functional groups [19,20,21]. Beyond other EM functional materials, graphene-based materials show advantages in the following aspects: (i) Excellent intrinsic properties, such as high conductivity and tunable dielectric permittivity: The 2D hexagonal packet structure ensures high charge carrier mobility that benefices construction of conductive networks via migrating and hopping electrons, endowing them with large conduction and scattering loss. (ii) The N-doping of graphene provides increased and rich surface chemical activity, making it easy to hybridize with other materials, effectively balancing strong EM loss ability and impedance matching as the most attractive absorbers. (iii) The well-designed hierarchical structure of graphene materials enables enhanced EM interactions and thus strong conductive and polarization loss [22]. Moreover, the remarkable properties of graphene, such as thermal conductivity, Young modulus, and inherent tensile strength, foster the production of multifunctional EM-shielding materials. The current types of graphene EM-shielding materials are mainly in the form of powders, coatings and aerogels [23]. It is to be noted that graphene-based EM functional materials have been mainly fabricated using graphene oxide (GO) or reduced rGO, requiring multistep, lengthy and complex processes, including use of reducing toxic agents. Hybridization of graphene with magnetic nanoparticles (e.g., Fe) was used as an effective way to break through the obstacle of impedance matching and high absorption losses [3]. An overview of the recent advances in EM functional materials can be found in recent reviews [23,24,25]. Although significant progress has been made in EM-shielding materials in recent years, challenges remain in achieving broadband absorption (e.g., across several microwave bands) and minimizing shield thickness. Moreover, the use of cumbersome, multistep batch processes remains a main concern regarding commercialization. Despite extensive efforts to investigate the influence of graphene-based building blocks on EM wave attenuation and energy conversion, the fundamental relationship between fine structural features and response dynamics under EM irradiation remains unclear, hindering precise control over EM performance. It is to be noted that EM wave attenuation performance critically hinges on fabrication methods, morphology, and hybridization ratio. It is essential to distinguish materials excelling in absorption vs. reflection, as shielding needs vary across EM environments. State-of-the-art approaches typically rely on simple melt-mixing of components (e.g., rGO, CNTs, and polymers), resulting in multistep batch processes that are often imprecise and lack cross-scale design control. Consequently, broadband electromagnetic absorption is generally achieved by varying shield thickness and filler loading, rather than through rational material design [23,24,25]. These limitations present significant challenges for graphene-based hybrid systems, hindering the realization of synergistic effects among their constituent components. Furthermore, effective electromagnetic shielding across multiple frequency bands often requires different material thicknesses and filler loadings for each band, which restricts practical implementation in increasingly congested electromagnetic environments where simultaneous shielding over a broad frequency range is essential. Optimally engineered nanocomposites that integrate dielectric and magnetic components with precise nanoscale design control offer a promising though technically demanding solution. The primary challenge lies in simultaneously achieving strong attenuation and effective impedance matching across a wide frequency range, which is essential for realizing the goal of “thin, lightweight, wideband, and high-performance” EM-shielding materials.

In this work, we present a plasma-enabled approach (Supplementary Information, Figure S1) for the controlled fabrication of ultralight nanocomposites with absorption-dominated electromagnetic interference (EMI)-shielding performance across an exceptionally broad frequency range (5–70 GHz). The approach employs a one-step plasma-driven assembly process to synthesize free-standing N-doped graphene (N-graphene) sheets uniformly decorated with metal oxide nanoparticles, forming hybrid nanostructures that are subsequently incorporated into a polymer matrix [26,27,28,29,30]. The microwave plasma environment simultaneously drives several key processes, including assembly of free-standing N-graphene nanosheets [29,30], nanoparticle generation from micron-sized precursors, surface functionalization, formation of multiple chemical phases, and nanoparticle attachment onto the graphene scaffold. These concurrent processes enable hierarchical nanostructure formation and precise control over molecular bond configurations and nanoscale interfacial interactions, thereby tailoring electromagnetic loss mechanisms. The method efficiently converts low-cost precursors, such as acetonitrile and micron-sized Fe_2_O_3_ and MnO_2_ particles, into N-graphene hybrid nanostructures at production rates up to 50 mg min^−1^, enabling gram-scale synthesis within minutes and rapid composite fabrication. The resulting nanocomposites exhibit absorption-dominated shielding, demonstrating more than 60% average absorption of incident microwave power across the 5–25 GHz range and exceeding 70% absorption in the 25–70 GHz region. Plasma-driven structural engineering at nanoscales, combined with multiphase hybridization, synergistically enhances dielectric and magnetic loss mechanisms, yielding effective absorption bandwidths (EABs), defined as the frequency ranges over which the reflection loss is below −10 dB, across key microwave bands, including C-band (2.1 GHz), Ku-band (3.4 GHz), Q-band (8 GHz), and V-band (4 GHz), at a minimum absorber thickness of 2 mm and constant filler content (15 *w%*).

## 2. Materials and Methods

### 2.1. Used Particle Precursors

Titanium(IV) oxide nanopowder: 21 nm primary particle size (TEM), >99.5% trace metals basis; Sigma-Aldrich (St. Louis, MO, USA).

Manganese(IV) oxide: activated, ~85%, <10 μm; Sigma-Aldrich (St. Louis, MO, USA).

Iron(III) oxide powder: <5 μm, <96%; Sigma-Aldrich (St. Louis, MO, USA).

### 2.2. Characterization Techniques

Transmission electron microscopy (TEM). The crystal structure and phase composition of the nanoscale materials were analyzed using a conventional transmission electron microscopy (TEM) instrument (JEM -2010F and JEM -2100, JEOL Inc., Tokyo, Japan) at 200 kV and a slow-scan CCD camera (ORIUS SC1000, Gatan Inc., Pleasanton, CA, USA). Scanning electron microscopy analyses were performed with a probe-corrected scanning transmission electron microscopy (STEM) instrument (ARM 200CF, JEOL Inc., Tokyo, Japan) operating at 200 kV and equipped with a high-angle dark-field (HAADF) detector (ADF and BF/DF detector, Gatan Inc. Pleasanton, CA, USA). The raw data were analyzed with Digital Micrograph software (Ver. 3.01, Gatan Inc.).

X-ray diffraction (XRD) measurements were carried out using a Bruker D2 Phaser (Bruker Inc., Oeiras, Portugal) diffractometer, equipped with a Cu source (CuKα λ = 0.154184 nm). The powdered samples were hand-pressed onto a zero-background sample holder (negligible background signal). The tests were made at room temperature, in the scan range of 2θ = 5 to 95°. Data were acquired with a step size of 0.03° and an integration time/step of 5 s (Supplementary Information, Figure S2).

Raman scattering. Inelastic photon scattering was performed by confocal Raman spectroscopy in back-scattering geometry, using He-Ne laser operating at 633 nm. In order to perform Raman spectroscopy characterization, the synthesized nanostructures were freely suspended on a glass substrate, and the Raman spectra from different regions of the substrate were obtained using a LabRAM HR Visible (Horiba Jobin-Yvon, Palaiseau, France) Raman spectrometer with 5 cm^−1^ spectral resolution and a laser spot size of 2 μm. Measurements were performed with a laser power P = 0.054 mW to avoid overheating.

X-ray photoelectron spectroscopy (XPS) measurements were performed on a VSW XPS system with the Class 100 energy analyzer being a part of an experimental setup (Multitécnica, VSW Instruments, Manchester, UK) assembled for surface investigation [31]. The survey and the high-resolution spectra were taken in fixed analyzer transmission mode with pass energy of 44 eV and 22 eV, respectively. The analysis was performed using the non-monochromatic Mg K_α_ line (photon energy of 1253.6 eV). For energy axis calibration, Ag (110) and polycrystalline Au samples (previously cleaned by ion sputtering) were used. The energy was calibrated to the peak position of the Ag 3d_5/2_ (binding energy of 368.22 eV) and Au 4f_7/2_ (binding energy of 83.96 eV) lines. The samples were analyzed as received, i.e., without any cleaning, to avoid bond breaking at the sample surface.

Dynamic light scattering (DLS) measurements were performed on a ZETASIZER Advance Pro (Malvern Panalytical, Worcestershire, UK), which measures particle and molecule size, from 0.3 nm to 10 µm (Supplementary Information, Figure S2).

Scattering parameters (S-parameters) were measured over a 5 to 70 GHz frequency range using two vector network analyzers (VNAs).

C-, X-, Ku- and-K bands were analyzed using a Rohde&Scharz ZNB VNA (Munich, Germany) [32], while Ka-, Q- and V-bands were analyzed using an Anritsu ME7838A Microwave VNA (Morgan Hill, CA, USA). The VNA ports were connected via suitable cables (e.g., ZV-Z193 [33] for the 5 to 26 GHz range) to band-specific sets of coaxial-to-waveguide adaptors (FMI FLAN Narrow Band Waveguide to SMA Coax Adaptors Series 094-SF40, Columbia, MD, USA [34]) and respective near-field probes (FLAN near-field probes Series 246, 100 mm in length, Cornwall, UK [35]). To ensure precise alignment and structural support, the system was mounted on custom 3D-printed holders and probe sleeves (see Supplementary Information, Figure S3). System calibration was performed for each waveguide frequency range using a Rohde&Scharz ZN-Z135 calibration kit in TOSM (Through-Open-Short-Match) mode [36] for the sub-26.5 GHz bands and an Anritsu 36585V-2M V precision Autocal for the remaining frequencies. Samples were prepared with dimensions of 2.5 × 4.5 cm^2^ and a thickness of 2 mm to ensure a precise fit across all waveguides. The filler loading was kept constant, i.e., 15 wt%. Sample thickness variation was kept below 0.1 mm to guarantee good measurement consistency.

## 3. Results and Discussion

An advanced microwave plasma-based approach enables the controlled, one-step, streamlined synthesis of free-standing N-doped graphene nanosheets decorated with chemically multiphase metal oxide nanoparticles under atmospheric-pressure conditions at the gram scale (Figure S1, Supporting Information). Leveraging microwave plasma’s ability to control quanta of energy and matter at nanoscales, an effective magnetic–dielectric synergy loss mechanism can be created. To this end, EM dissipation can be managed via control of dipole density (e.g., nitrogen atoms in pyridinic, pyrrolic and other functional groups attached to the graphene scaffold acts as dipoles) and magnetic/dielectric nanoparticle size and distribution over graphene nanosheets (e.g., tuning density and phases of Fe_x_O_y_ nanoparticles, natural and exchange resonances, etc.). The attached functional groups in N-graphene as pyridinic, pyrrolic and defects strongly affect dipole relaxation losses as far as functional groups, defects and interfaces behave as dipoles. These dipoles will rotate in an EM field, and when they are unable to follow EM field changes, relaxation loss takes place. Incorporating TiO_2_ nanoparticles can reduce the high dielectric constant of the carbon scaffold, thereby improving the impedance-matching characteristics and enhancing the contribution of interfacial polarization to dielectric losses. In contrast, the incorporation of MnO_2_ nanoparticles may further increase eddy-current losses in addition to interfacial polarization losses [37,38]. Moreover, the excitation of localized surface plasmon resonances on the N-doped graphene scaffold may also contribute to the overall electromagnetic loss mechanisms [30]. As a precursor of N-graphene, acetonitrile, serving as a single-source precursor for both carbon and nitrogen, was injected in a top-down configuration directly into the hot plasma zone, enabling the direct synthesis of N-doped graphene sheets (see Figure S1, Supplementary Information) [28]. To demonstrate the mastering of dielectric–magnetic synergy loss mechanisms, two different combinations of metallic oxide precursors were used to decorate free-standing N-graphene nanosheets with nanoparticles. The first combination of nanoparticle precursors includes a mixture of magnetic micron-size Fe_2_O_3_ (50%) particles and TiO_2_ (50%) nanoparticles. The second one is composed of Fe_2_O_3_ (70%) and MnO_2_ (30%). A precisely controlled jet of micron-size particles mixtures was introduced upstream into the “mild” plasma region to decorate N-graphene nanosheets. The carrier argon gas was introduced in swirl injection mode to enhance gas-phase mixing and stabilize the flow dynamics within the reactor (Figure S1).

### 3.1. Material Characterization of N-Graphene-Fe_2_O_3_-TiO_2_ (NG-F-T) Hybrids

**Figure 1:**
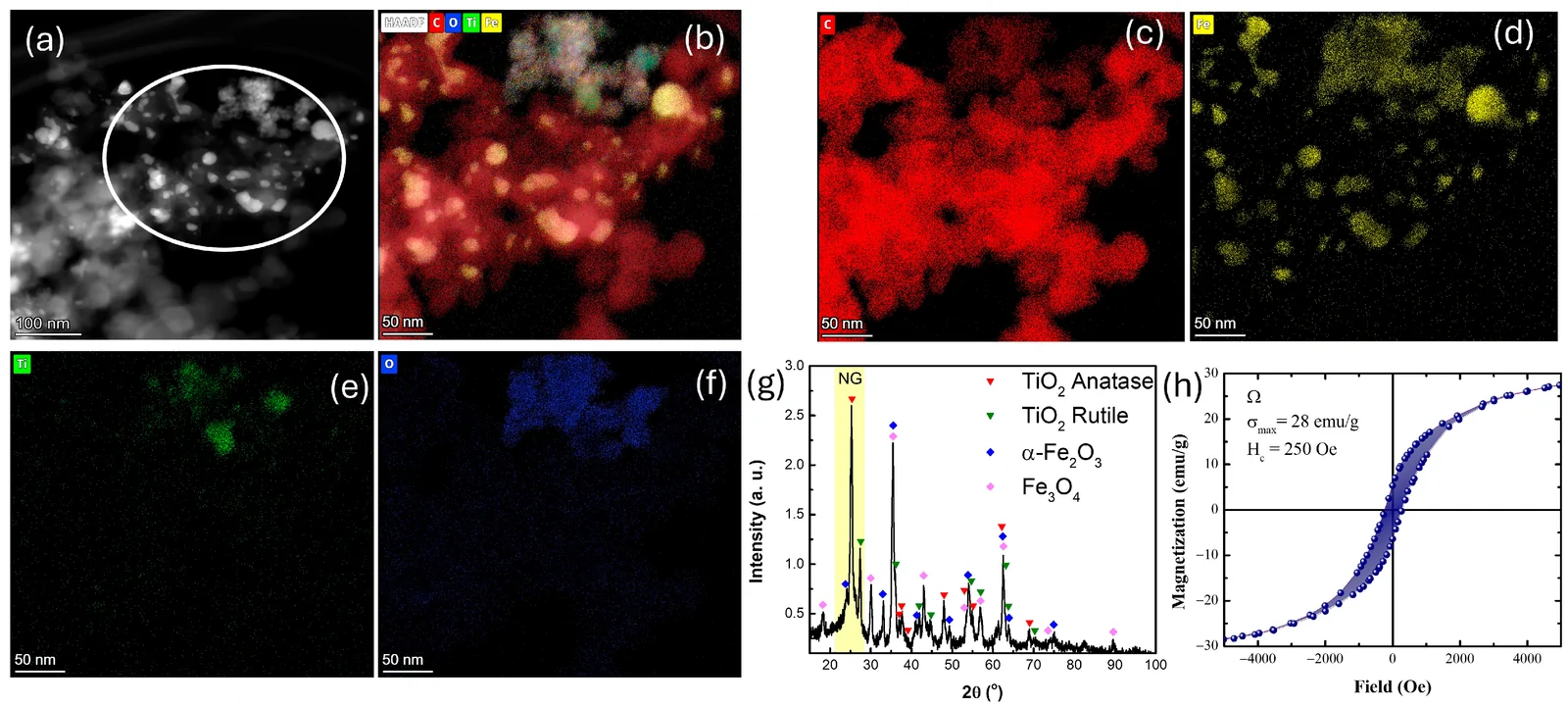
(**a**) HAADF-STEM micrograph of NG-F-T hybrid; (**b**) overlay of elemental mapping; (**c**–**f**) spatial distribution of principal components, C, Fe, Ti, and O, by EDS; (**g**) XRD pattern of NG-F-T hybrid; (**h**) magnetic hysteresis curve.

The surface morphology and structural organization of the nanocomposites were studied by STEM. High-angle-annular dark-field (HAADF) STEM micrographs of the nanocomposite (Figure 1a) reveal graphene-like sheets decorated with nanoparticles of varying sizes, ranging from a few nm to ~ 50 nm. The corresponding energy-dispersive X-ray spectroscopy (EDS) elemental analysis (Figure 1b–f) confirms the presence of Fe- and Ti-oxide nanoparticles attached to graphene sheets.

The phase composition of the material was assessed by XRD. The precursors introduced were TiO_2_ anatase (COD 9008213), rutile (COD 9004142), and the γ-Fe_2_O_3_ phase (PDF 39-1346) (Figure S2a; Supplementary Information). The XRD pattern confirms the successful formation of hybrids (Figure 1g), with high intensity and a wide peak at 2θ ~ 25.81° (002), designated as NG and overlapped with the TiO_2_ anatase (PDF 21-1272) phase, indicating the graphene-based nature of the material, as well as various newly formed nanocrystalline phases. The XRD pattern of the NG-F-T hybrid confirms the successful formation of new nanocrystalline phases of attached metal nanoparticles, different from those of the precursors (Figure 1g), identified as α-Fe_2_O_3_ (PDF 33-0664) and Fe_3_O_4_ (PDF 19-0629). The precursor chemical phases of TiO anatase (COD 9008213) and rutile (COD 9004142) are also present. It is to be noted that the XRD patterns of the precursors demonstrate the presence of only the γ-Fe_2_O_3_ phase (PDF 39-1346) (Figure S2a; Supplementary Information). It is to be noted that the γ-Fe_2_O_3_ phase is a metastable phase and transforms into α-Fe_2_O_3_ at high temperatures (typically in the range 250–750 °C). Considering that the temperatures in the “mild” plasma zone are in the range of 300–1000 K, it is very likely that the γ-Fe_2_O_3_ phase is transformed into α-Fe_2_O_3_. The formation of the Fe_3_O_4_ (PDF 19-0629) phase is due to the reduction of the α-Fe_2_O_3_ phase.

Additionally, the magnetic properties of the synthesized sample were investigated. The magnetization-versus-magnetic field hysteresis curves were measured at room temperature by a Vibrating Sample Magnetometer (VSM) up to a maximal magnetic field of 6 kOe. The samples were prepared by pressing the NG-F-T hybrid powder into cylindrical quartz containers so that the particles could not move during the measurements. Ferromagnetic behavior is evident from the magnetic hysteresis curve, shown in Figure 1g. For the nanocomposites investigated, saturation magnetization ($\sigma_{\max}$) and coercivity ($H_{c}$) were determined to be 28 emu/g and 250 Oe, respectively.

**Figure 2:**
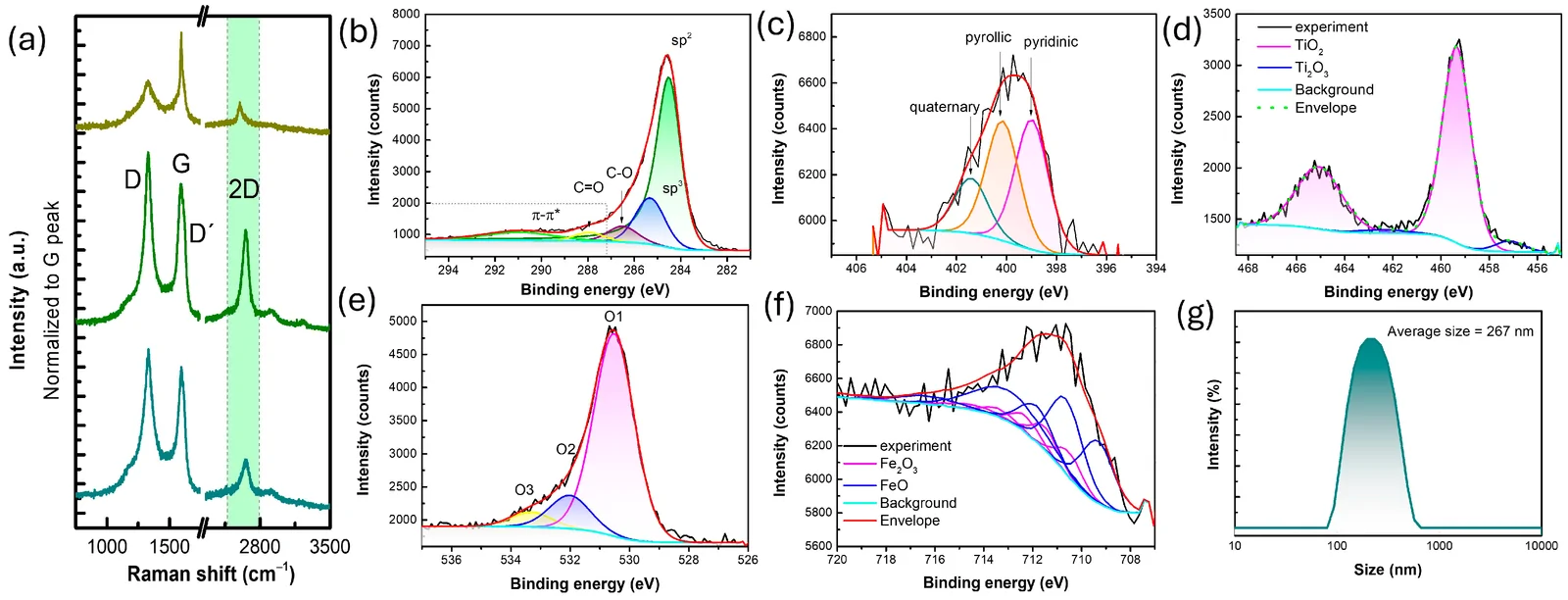
(**a**) Raman spectra; (**b**–**f**) XPS C 1s, N 1s, Ti 2p, O 1s, and Fe 2p_3/2_ regions respectively; (**g**) size distribution of NG-F-T hybrid particles.

The Raman spectra collected from four randomly selected spots of the nanocomposite sample are presented in Figure 2a. The characteristic D-, G-, and 2D-bands typical of graphene-like materials are observed, although some regions exhibit a notably low-intensity 2D-peak. The presence of nanoparticles attached or intercalated to the graphene sheets as seen from STEM images results in the suppressing of 2D-peaks, which results from the two-photon resonance process in pure graphene. The presence of D-peaks is directly related to the type and concentration of defects in the graphitic structure. The pronounced D-band intensity therefore reflects both the incorporation of nanoparticles and nitrogen doping in the graphene lattice. Moreover, the edges of nanohybrids, “seen” as defects, also contribute to D-peak intensity [39,40].

Dynamic light scattering data demonstrate hybrids nanostructures with an average size of about ~270 nm (Figure 2g), while the average size of the Fe_2_O_3_ precursor particles is about 1 μm (Figure S2b).

The NG-F-T surface structures were further analyzed for surface chemical composition using X-ray photoelectron emission spectroscopy (XPS). The composition of samples was determined from the intensities of the characteristic lines of the detected elements, using the relative sensitivity factors from [41]. High-resolution spectra of the C 1s, N 1s, O 1s, Ti 2p and Fe 2p_3/2_ photoelectron lines are presented in Figure 2b–f. The results from quantitative composition analysis are shown in Table 1.

As expected, the graphene scaffold is primary composed of carbon, nitrogen and oxygen. The C 1s line mainly consists of pure graphene (at 284.5 eV), contributing about 61% of the total C 1s line intensity (Figure 2b). Minor contributions from sp^3^-hybridized carbon or saturated hydrocarbons (C-C and C-H bonds) were also observed, representing about 20% of total intensity. C-N bonds are likely included within this contribution. The remaining carbon atoms are attributed to oxygen-containing functional groups, specifically C-O (6.9%) and C=O bonds (3.5%). The contribution of π–π* resonances is about 8.1%.

The fitting of the N 1s line, presented in Figure 2c, was performed by introducing three symmetric contributions with the same width. They could be attributed to pyridinic, pyrrolic and quaternary nitrogen. The following should be kept in mind: Instead of pyridinic, it may be considered a 2-coordinated nitrogen bonded to sp^2^-hybridized carbon atoms. Similarly, pyrrolic can be generalized into 3-coordinated nitrogen that can have sp^2^-hybridized carbon atoms as neighbors. As a result, for every “pyridinic” nitrogen, there is the contribution from two sp^2^ carbon atoms, and for every pyrrolic nitrogen, there is the contribution of two or three carbon atoms. These contributions should be situated in the range 285–287 eV; i.e., they should be found in the sp^3^ and C-O energy ranges. Consequently, a reduced intensity of the sp^2^ peak could be expected, while parts of the sp^3^ and C-O peaks actually correspond to N-doped graphene, i.e., to the carbon atoms from graphene bonded with N. The quaternary contribution in the C 1s line should be presented at binding energies above 287 eV, that is, in the C=O peak. Knowing that only 0.5% of all detected atoms are considered to be quaternary nitrogen, the contribution in C 1s should also be minor.

The major metallic lines of the oxides, i.e., Ti 2p and Fe 2p_3/2_, taken from the composite sample are presented in Figure 2d,f [42,43]. In the case of titanium, the dominant TiO_2_ phase is accompanied by the minor Ti_2_O_3_ phase (about 5.1%). Knowing that the relative amounts of Ti atoms in the TiO_2_ and Ti_2_O_3_ phases are 33% and 40%, respectively, and after considering the Ti atomic concentrations in the two phases (3.19 × 10^22^ and 3.76 × 10^21^ cm^−3^, respectively), the actual ratio of the two phases (i.e., the ratio of the corresponding molecular concentrations), TiO_2_:Ti_2_O_3_, can be roughly estimated as 98:2. Although small, we do observe a reduction of the oxide phase on the surface of the sample under analysis.

The same and more pronounced trend can be observed in the case of the iron oxide phase (Figure 2f). The ratio of FeO:Fe_2_O_3_ intensity grew from 66:33 to 78:22, which means that the iron oxide phase generally decreases its oxidation state. It should be noted that an equivalent analysis of the ratio of concentrations of different oxide phases was not performed, since it may be misleading due to the presence of the Fe_3_O_4_ phase, which can be also interpreted in XPS as a mixture of the FeO and Fe_2_O_3_ phases. It should be noted that the data are relative to the surface of the sample.

The oxygen O 1s photoelectron line, presented in Figure 2e, is consistent with the metallic lines: the most dominant peak, O 1s, is shifted by about 0.9 eV with respect to the expected position for different lines. The O_2_ peak corresponds to the defective surface oxygen of metallic oxides, having the same shift due to charging. The O_3_ peak should be organic oxygen, which does not have charging (knowing that C 1s was not charging) [44].

### 3.2. Material Characterization of N-Graphene-Fe_2_O_3_-MnO_2_ (NG-F-M) Hybrids

**Figure 3:**
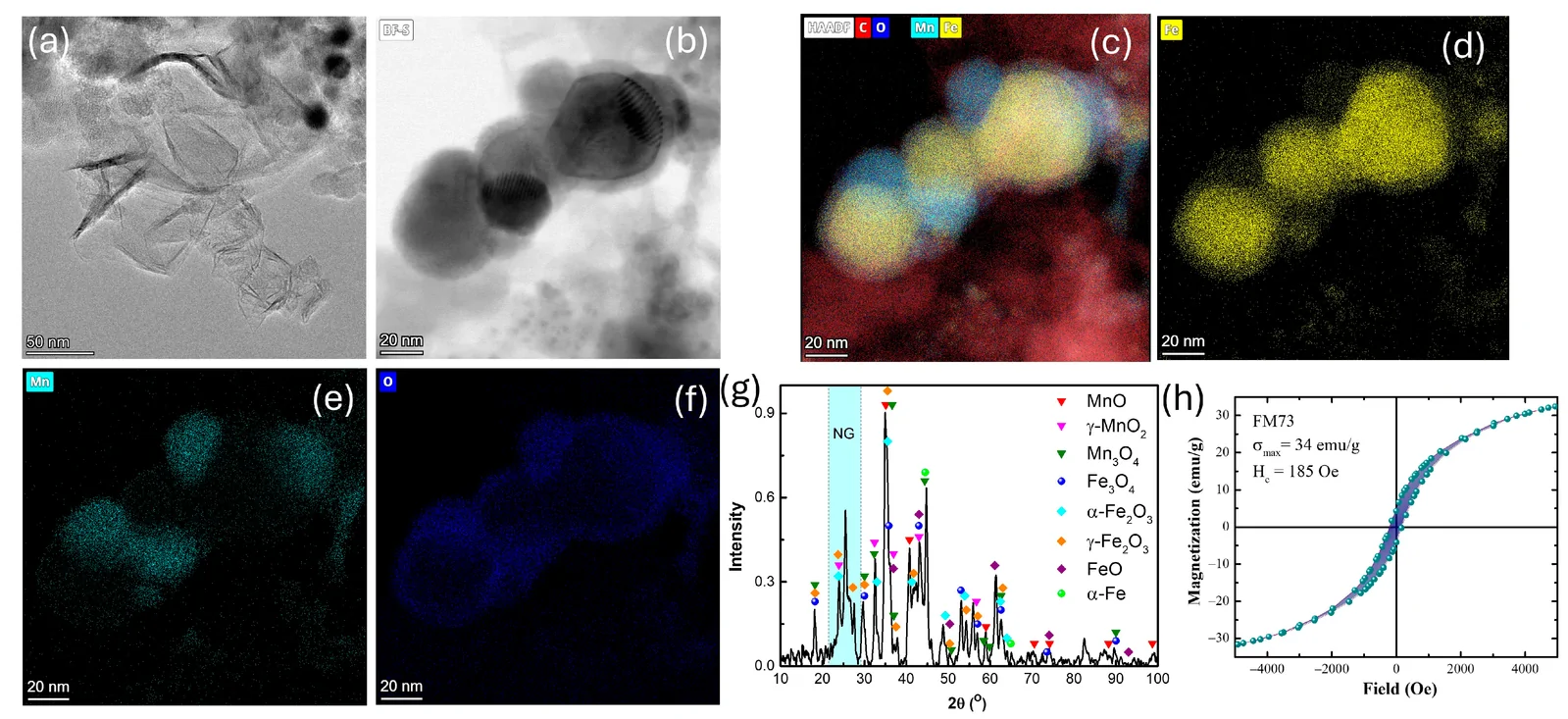
(**a**) HRTEM micrograph of the hybrid material; (**b**) HAADF-STEM micrograph of the NG-F-M hybrid; (**c**) STEM-EDS overlay; (**d**–**f**) EDS spatial distribution of Fe, Mn, and O; (**g**) XRD pattern; (**h**) magnetic hysteresis curve.

HRTEM and high-angle-annular bright-field (HAABF) STEM images of the nanocomposite (Figure 3a,b) reveal graphene-like sheets decorated with nanoparticles of varying sizes ranging from 5 to ~ 60 nm. The corresponding EDS elemental analysis (Figure 3c–f) confirms the presence of Fe and Mn nanoparticles on transparent graphene sheets within the synthesized structure. The results clearly demonstrate the reduction in particle size from micron-size to nano-size (Supplementary Information, Figure S2).

The XRD pattern of the synthesized N-graphene nanocomposite (NG-F-M) displays multiple peaks, confirming the presence of various new-born nanocrystalline phases (see Figure 3g). It shows the typical planes attributed to graphitic (002) planes (marked NG in the figure) at 2θ ~ 25.7° and to the cubic Fm3m MnO phase (PDF#07-0230; a = 0.4445 nm). Minor diffraction peaks are assigned to γ-MnO_2_ (COD 1514239) and the α-Mn_3_O_4_ phase (PDF #24-0734; a = 0.5762 nm), respectively. The dominant crystalline phases of iron oxide identified are γ-Fe_2_O_3_ (PDF 25-1402), α- Fe_2_O_3_ (COD 9000139) and Fe_3_O_4_ (PDF 19-0629). Additionally, some minor diffraction peaks are identified as the α-Fe phase (PDF 06-0696) and FeO (COD 1011198). The plasma chemical reactivity resulted in modification of the metal microparticles and the creation of new metallic phases (Supplementary Information, Figure S2a,c).

The magnetic hysteresis loop of the NG–F–M hybrid is shown in Figure 3h. Its measured maximum magnetization (σ_max_) and coercivity (H_c_) are 34 emu/g and 185 Oe, respectively, compared with 28 emu/g and 250 Oe for NG–F–T. Thus, NG–F–M exhibits higher maximum magnetization and lower coercivity. The magnetization values are normalized to the total mass of each hybrid, including the N-graphene component, and therefore represent the overall magnetic response of the hybrid materials.

XRD analysis reveals distinct crystalline phase assemblages in the two hybrids. NG–F–T contains α-Fe_2_O_3_ and Fe_3_O_4_, whereas NG–F–M additionally contains γ-Fe_2_O_3_, metallic Fe, and FeO, alongside the Mn-containing phases MnO, γ-MnO_2_, and α-Mn_3_O_4_. The higher maximum magnetization of NG–F–M is consistent with its different Fe-containing phase assemblage, particularly the presence of metallic Fe and ferrimagnetic iron oxides. Its lower coercivity may also reflect differences in phase composition and microstructure. These observations support interpreting the magnetic behavior in terms of the combined response of the constituent phases and their microstructural environment, rather than attributing the differences exclusively to the Mn-containing phases.

**Figure 4:**
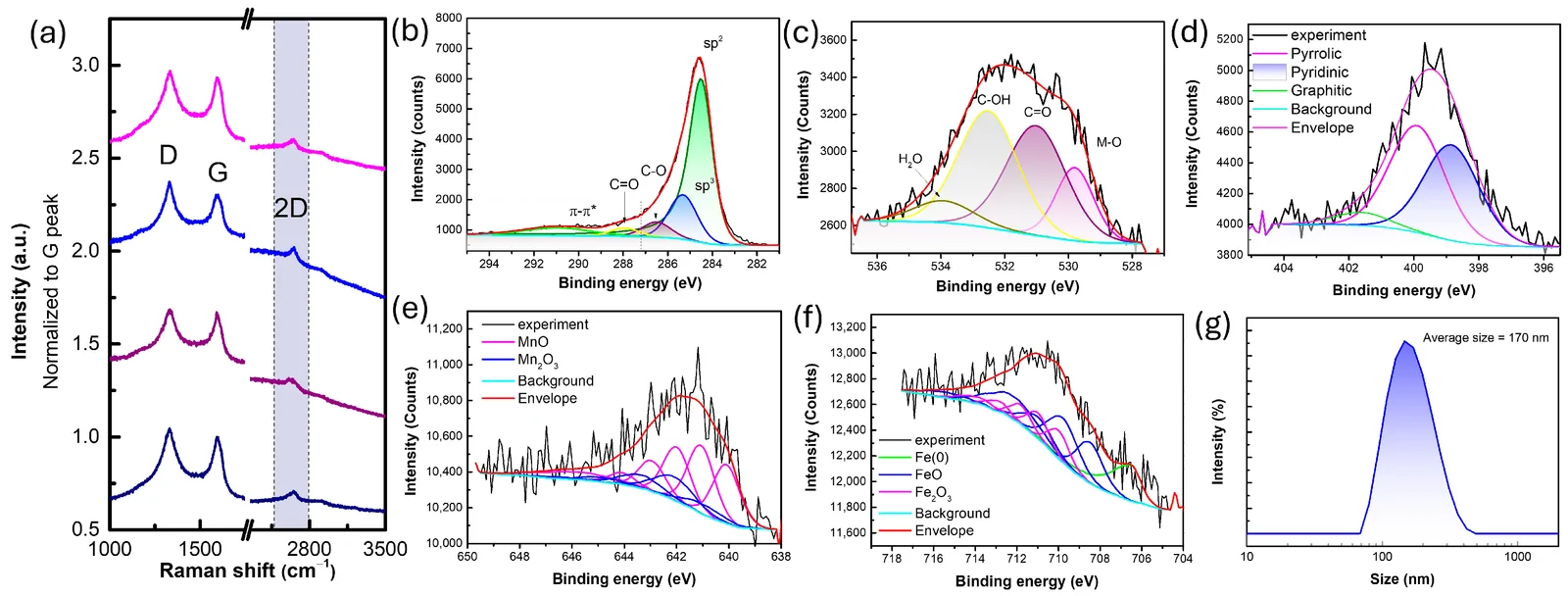
(**a**) Raman spectra; (**b**–**f**) XPS C 1s, O 1s, N 1s, Mn 2p_3/2_, and Fe 2p_3/2_ regions respectively; (**g**) size distribution of NG-F-M hybrid particles.

The Raman spectra acquired from three randomly selected locations of the sample exhibit the characteristic D-, G- and 2D-bands associated with graphene-like materials (Figure 4a). The 2D-peak intensity is much smaller as compared with D- and G-peaks. The pronounced D-band intensity across all spectra suggests high density of defects, likely arising from nitrogen incorporation into the graphene lattice and/or the anchoring of nanoparticles onto the graphene surface.

The NG-F-M structures are further analyzed for surface chemical composition using XPS spectroscopy. High-resolution spectra of the C 1s, N 1s, O 1s, Mn 2p_3/2_ and Fe 2p_3/2_ photoelectron lines are presented in Figure 3b–f.

As expected, the graphene scaffold consists of carbon, nitrogen and oxygen, while iron, manganese and parts of O partake in the iron manganese oxide phases. The C 1s line mainly consists of pure graphene (typically at 284.3 eV), contributing about 73.0% of the total C 1s line intensity [45,46]. Considerable quantities of sp^3^ carbon or saturated hydrocarbons (C-C and C-H bonds), which cannot be reliably separated due to the proximity of their contributions, were also detected (about 20.9%). It is expected that C–N bonds also contribute to this feature, which may account for its relatively high intensity. The remaining carbon atoms are bonded with oxygen in C-O and C=O bonds. The peak positions of the C-O bonds were identified according to [47] and adjusted so that aliphatic saturated hydrocarbons were situated at 284.8 eV. The N 1s line was fitted to three contributions, which can be attributed to pyridinic N (at 398.8 eV, 44.3%), pyrrolic N (at 399.9 eV, 49.6%) and graphitic N (at 401.6 eV 6.1%) [47]. However, one should note that the “amine” contribution is also very close to the pyrrolic contribution, expected at 399.9–400.7 eV [43] (amine is expected in the range 399.6–399.9 eV [47]).

Since the shapes of the main photoelectron lines of Fe (Fe 2p_3/2_) and Mn (Mn 2p_3/2_) are complex due to the multiplet splitting effect, they were fitted to a set of peaks interrelated with well-defined constraints [43]. Therefore, although there are up to five peaks per one phase, the actual number of completely free parameters per phase is only two: the position and the intensity of the first line. About 75.5% of the Mn 2p_3/2_ line corresponds to the MnO phase, while the remaining 24.5% is Mn_2_O_3_ (Figure 4e). According to the fractions of Mn atoms bonded in the MnO phase and the Mn_2_O_3_ phase, the estimation shows that 86.1 mol% of manganese oxide corresponds to MnO, while the rest is Mn_2_O_3_.

From the fitting of the Fe 2p_3/2_ line, it appears that 17.2% of the detected iron atoms correspond to metallic iron, 57.1% originate from the FeO phase and the remaining 25.7% are from the Fe_2_O_3_ phase (Figure 4f). The data are in accordance with the XRD analysis. As for the molar fractions of the iron oxide phase, it consists of 81.6 mol% FeO and 18.4 mol% Fe_2_O_3_, and the Fe:O concentration ratio in the iron oxide phases is about 48:52.

Fitting of the O 1s line enabled us to disentangle the metallic oxide contribution from the C-O bonds and the adsorbed water contribution. The narrow peak at about 529.8 eV corresponds to Fe-O and Mn-O bonds (Figure 4c). The wider peaks at 531.0 eV and 532.5 eV were attributed to C=O bonds in the aromatic scaffold and aliphatic C-O-C bonds, respectively [44]. Finally, a small contribution at 533.9 eV corresponds to adsorbed water.

Dynamic light scattering data demonstrate a significant reduction in particle size. The NG-F-M hybrid particles’ average size is around 170 nm (Figure 4g), while precursor particles are micron-size (Supplementary Information, Figure S2d).

### 3.3. Electromagnetic Performance

**Figure 5:**
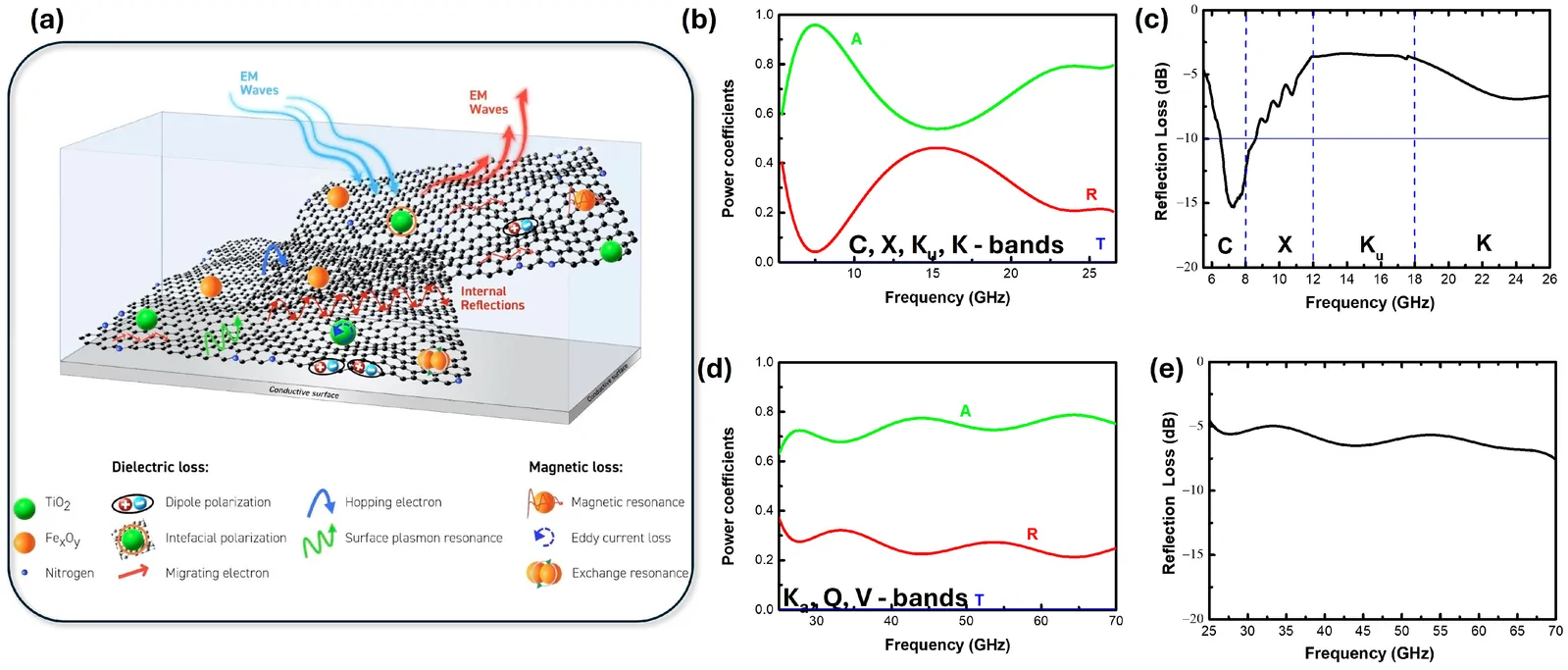
(**a**) Main dielectric and magnetic loss mechanisms. (**b**,**c**) Power coefficients and reflection loss in 5–26 GHz frequency range and (**d**,**e**) power coefficients and reflection loss in 25–70 GHz frequency range of the NG-F-T-epoxy nanocomposite.

The synthesized N-graphene hybrids (NG-F-T; NG-FM) were blended with epoxy resin in a filler loading ratio 15 *wt%*. To evaluate performance in application-relevant configurations, the NG-F-T-Epoxy and NG-F-T-Epoxy nanocomposites were deposited onto conductive substrate with a minimal coating thickness of 2 mm (Figure 5a).

When an electromagnetic (EM) wave impinges on the surface of a shielding material, the free electrons on the surface generate an opposing electric field that counteracts the incident EM wave. This phenomenon is associated with impedance mismatch between free space and the shielding material, causing a portion of the incident wave to be reflected. The remaining EM waves penetrate into the material, where they are converted into thermal energy through different dielectric and magnetic loss mechanisms. The EM waves that are not immediately absorbed undergo multiple internal reflections between the two surfaces of the shielding material, resulting in continuous attenuation before eventual transmission or dissipation. Achieving maximum electromagnetic absorption while minimizing reflection over a broad frequency range requires the simultaneous optimization of strong electromagnetic wave attenuation and effective impedance matching. Dielectric losses arise from multiple mechanisms, including conduction losses associated with electron hopping and migration, dipolar and interfacial polarization, and localized surface resonances. Magnetic losses, in turn, originate from natural and exchange resonances, as well as eddy-current effects (Figure 5a). The synergistic interplay between these dielectric and magnetic loss mechanisms is therefore essential for enhancing electromagnetic energy dissipation while maintaining effective impedance matching with free space. Such synergy enables strong attenuation of incident EM waves while suppressing reflected power, ultimately promoting broadband, absorption-dominated electromagnetic shielding.

The waveguide method was employed to measure the scattering (S) parameters [48]. In this method, the signal generated by the vector network analyzer (VNA) is transmitted through a waveguide fixture, as illustrated in Figure S3 (Supplementary Information). Photographs of the synthesized N-graphene nanocomposites and their corresponding epoxy-embedded composites are also presented in Figure S3 (Supplementary Information).

In this study, the electromagnetic shielding efficiency of nanocomposites was investigated over the 5–70 GHz frequency range, covering seven microwave bands: C, X, Ku, K, Ka, Q and V. Two nanocomposite systems with different chemical compositions of N-graphene hybrids (15 *w%*) embedded in an epoxy polymer matrix were examined: N-graphene decorated with a mixture of iron oxide and manganese oxide phases (NG-F-M) and N-graphene decorated with Fe_x_Oᵧ/TiO_2_ nanoparticles (NG-F-T).

A clear description of the shielding performance can be obtained using the power coefficients of transmissivity (T), reflectivity (R), and absorptivity (A), which satisfy the following relation:

T+R+A=1

where $T=\frac{P_{t}}{P_{i}},\;R=\frac{P_{r}}{P_{i}},\;A=\;\frac{P_{a}}{P_{i}}$ are calculated using scattering (S-)parameters [48]. Here, $P_{t}$, $P_{r}$, and $P_{a}$ represent the transmitted, reflected, and absorbed power of the incident electromagnetic wave, respectively, while $P_{i}$ is the incident power.

Furthermore, reflection loss (RL) and the EABs were derived using transmission line theory [3,49,50,51,52]. Reflection loss is a measure of the fraction of incident microwave power reflected back from the shielding material rather than being absorbed or transmitted. It is widely used to evaluate the microwave absorption performance of materials and is calculated as:

$$RL\left(dB\right)=20\log|\frac{Z_{in}-Z_{0}}{Z_{in}+Z_{0}}|$$

where $Z_{in}$ is the normalized input impedance of the shielding material and $Z_{0}$ is the impedance of free space. A more negative RL value indicates better impedance matching and stronger microwave absorption performance.

Figure 5 presents the frequency-dependent reflection (R), transmission (T), and absorption (A) power coefficients of the NG-F-T-Epoxy nanocomposite over the 5–70 GHz frequency range. As shown in Figure 5b, the material exhibits absorption exceeding 55% across four frequency bands within the 5–27 GHz range at a minimum thickness of 2 mm, as previously noted. The absorption increases significantly in the C- and K-band regions, reaching up to 90% in the C-band (5–8 GHz), while reflection remains below 50%. In contrast, impedance mismatch is observed in the X- and Ku-bands, where reflection approaches nearly 50%. A second absorption maximum, corresponding to approximately 80% absorbed power, is observed in the K-band. Reflection losses below −10 dB, corresponding to more than 90% absorption, indicate an effective absorption bandwidth of approximately 2.1 GHz in the C-band (Figure 5c).

Across three microwave bands within the 25–70 GHz frequency range, the absorptance remains nearly constant, with absorption efficiency exceeding 70% in the Ka and V-bands.

**Figure 6:**
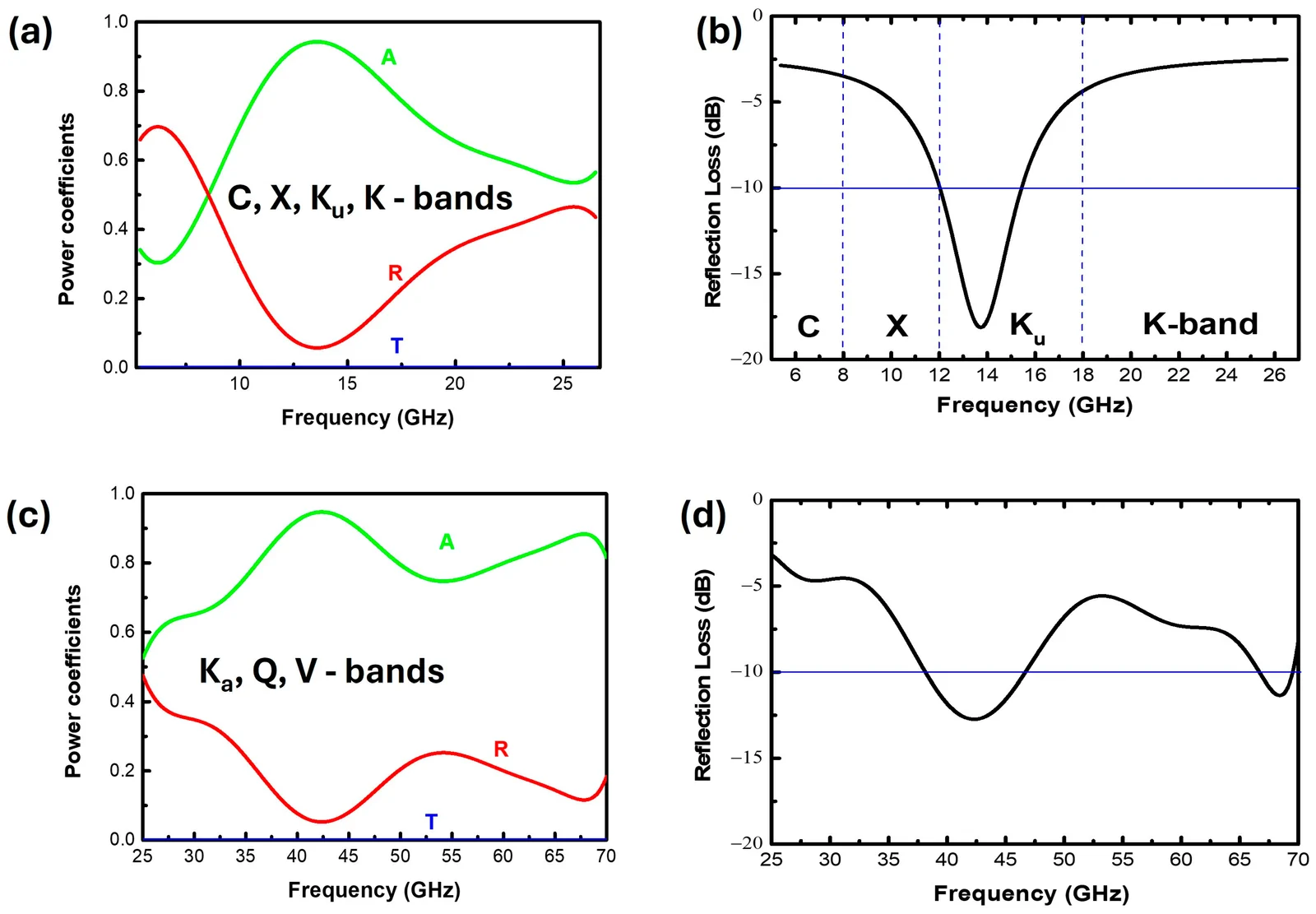
(**a**,**b**) Power coefficients and reflection loss in the 5–26 GHz frequency range and (**c**,**d**) power coefficients and reflection loss in the 25–70 GHz frequency range of the NG-F-M-epoxy nanocomposite.

The NG-F-M-Epoxy nanocomposite exhibits average absorption exceeding 60% over the 8–26 GHz frequency range. As shown in Figure 6a, absorption increases significantly in the X-, Ku-, and K-band regions, reaching up to 90% in the Ku-band, while reflected power remains below 50%. In contrast, impedance mismatch is observed in the C-band, where reflection (~60%) is approximately twice the absorption, and reflection remains close to 50% at higher frequencies in the K-band, indicating less effective impedance matching in these regions. Reflection losses below −10 dB indicate an EAB of approximately 3.4 GHz in the Ku-band (Figure 6b). 

A notable result is that absorption efficiency exceeds 70% in the K- and V-bands (35–70 GHz). In addition, enhanced absorption bands with EAB values of approximately 8 GHz in the K_a_-band and 4 GHz in the V-band are observed, confirming the excellent microwave absorption performance of this nanocomposite even at the small shield thickness of 2 mm.

To demonstrate the synergistic interplay between dielectric and magnetic loss mechanisms and their combined contribution to electromagnetic wave absorption, the key electromagnetic parameters of the investigated nanocomposites were analyzed in the Ku-band frequency range (12–18 GHz). This frequency range is particularly relevant, as it encompasses two contrasting regimes of electromagnetic response, characterized by minimum (Figure 5b,c) and maximum (Figure 6a,b) absorption.

The imaginary part of complex permittivity, ε″, which describes the material’s ability to dissipate electric field energy, exhibits a pronounced contribution relative to the real part, ε′, indicating strong dielectric losses and contributing to the predominance of absorbed over reflected power (Supplementary Information, Figures S4a and S6a). A similarly pronounced frequency-dependent behavior is observed for the real (μ′) and imaginary (μ″) components of complex magnetic permeability (Supplementary Information, Figures S4b and S6b), confirming the contribution of magnetic loss processes to the overall EM wave attenuation.

The occurrence of negative values of the real part of dielectric permittivity, ε′, suggests that in addition to dipolar and interfacial polarization losses, local plasmonic-type resonances induced by the interaction of EM waves with the conductive N-graphene nano-islands and metallic nanoparticles may also contribute to the overall dielectric loss. This behavior of the dielectric permittivity is consistent with our previous observations [30].

Compared with the NG-F-T-Epoxy nanocomposite, NG-F-M-Epoxy exhibits stronger attenuation and higher AC conductivity, resulting in more efficient dissipation of incident microwave energy and absorption exceeding 80% within the Ku-band (Supplementary Information, Figures S5a–c and S7a–c). In addition, the impedance of NG-F-M-Epoxy is better matched to that of free space, with the normalized impedance approaching unity. This improved impedance matching facilitates EM wave penetration into the material and suppresses reflection, resulting in reflected power below 20% (Supplementary Information, Figures S5c and S7c).

Further insight into the magnetic loss mechanisms is obtained from the frequency dependence of the eddy-current coefficient, C_0_ = μ″/(μ′^2^f) (see Supplementary Information). Its variation with frequency **f** (Supplementary Information, Figures S5d and S7d) indicates that natural and exchange resonances are the dominant contributors to magnetic losses over most of the Ku-band. In contrast, the contribution from eddy-current losses becomes more significant for the NG-F-M-Epoxy nanocomposite in the 12–14 GHz range, where C_0_ approaches a nearly frequency-independent value.

Consequently, the superior microwave absorption performance of NG-F-M-Epoxy in the Ku-band originates from the synergistic interplay of multiple dielectric and magnetic loss mechanisms, including conduction losses, dipolar and interfacial polarization, surface resonances, magnetic resonances, and eddy-current losses, combined with effective impedance matching that facilitates the efficient coupling and dissipation of incident electromagnetic energy.

The obtained results demonstrate excellent electromagnetic wave absorption across a broad frequency range of 65 GHz, particularly given the small absorber thickness of only 2 mm. It should be noted that increasing the shield thickness would further enhance the absorption performance over this frequency range.

## 4. Conclusions

In this study, plasma-enabled strategy for the controllable fabrication of ultralight nanocomposites with absorption-dominated EM-shielding performance in a very large 5–70 GHz frequency range is presented. The fabricated nanocomposites achieve average microwave absorption levels above 60% across the 5–25 GHz frequency range and 70% in the 25–70 GHz range, at a constant shield thickness of 2 mm and a filler loading of 15 *wt%*. In contrast to conventional chemical approaches, where broadband absorption is typically achieved by varying shield thickness and filler loading, the present plasma-assisted strategy enables dominant absorption over a wide frequency range without altering these parameters. The proposed approach employs a one-step plasma-driven assembly process to synthesize N-graphene sheets uniformly decorated with metal oxide nanoparticles, forming N-graphene hybrid nanostructures that are subsequently incorporated into a polymer matrix. Plasma-driven nanoscale engineering combined with multiphase hybridization synergistically enhances both dielectric and magnetic loss mechanisms, resulting in effective absorption bandwidths spanning the C-band (2.1 GHz), Ku-band (3.4 GHz), K_a_-band (8 GHz), and V-band (4 GHz). The plasma process facilitates the formation of free-standing nitrogen-doped graphene (N-graphene) sheets uniformly decorated with metal oxide nanoparticles (Fe_x_O_y_, MnO_x_, and TiO_x_), which are subsequently embedded within a polymer matrix to fabricate the nanocomposite shielding material.

Overall, the proposed plasma-enabled synthesis offers a scalable and efficient route for engineering multifunctional lightweight nanocomposites with tunable electromagnetic properties while eliminating the need for complex, time- and energy-intensive multistep chemical processing, a major barrier to commercialization. The streamlined methodology enables precise control over material composition, morphology, and interfacial characteristics, facilitating clear structure–property relationships. Furthermore, it provides a versatile platform for the large-scale production of next-generation lightweight EM-shielding materials, with strong potential to improve manufacturing efficiency, reduce costs, and shorten processing times for advanced electronic and aerospace applications.

## 5. Patents

Process, (Reactor) and System production 2D nanostructures, INPI, PT 109387 B, 14 December 2021; Process, (Reactor) and System production 2D nanostructures, EPO, EP3455389, 24 May 2024; Process, (Reactor) and System production 2D nanostructures, USPTO, US 11,254,575 B2, 22 February 2022; Process, (Reactor) and System production 2D nanostructures, JPO, JP2019523695, 1 August 2022; Reactor production 2D nanostructures (divisional), INPI, PT 117332, 12 May 2022; Process, Reactor, System customization nanostructures, INPI, PT 110764 B, 24 November 2021; Process production nanocomposites, INPI, PT 115782 B, 2021–11–04.

## Figures and Tables

**Table 1:** Quantitative composition analysis of the samples.

NG-F-T	C	O	N	Ti	Fe
	77.7	14.4	2.9	3.6	1.4

## Data Availability

The original contributions presented in this study are included in the article/Supplementary Materials. Further inquiries can be directed to the corresponding authors.

## Supplementary Materials

The following supporting information can be downloaded at: https://www.mdpi.com/article/10.3390/nano16211358/s1. Figure S1: Schematic representation of the plasma reactor for free-standing N-graphene nanosheets and N-graphene hybrids synthesis. High-angle-annular dark-field (HAADF) STEM image of NG-F-T and NG-FM hybrids and EDS images; Figure S2: (a,b,c) XRD pattern of Fe_2_O_3_ MnO_2_ and TiO_2_ precursors, respectively; (d,e) DLS of Fe_2_O_3_ and MnO_2_ precursor particles; Figure S3: Experimental setup for measurements of scattering S-parameters; Figure S4: Real and imaginary parts of dielectric permittivity and magnetic permeability of NG-F-T Epoxy nanocomposite; Figure S5: Electrical conductivity σ_ac_(a), attenuation constant α(b), impedance matching Z(c) and Eddy current loss C_o_(d); Figure S6: Real and imaginary parts of dielectric permittivity and magnetic permeability of NG-F-M-Epoxy nanocomposite; Figure S7: Electrical conductivity σ_ac_ (a), attenuation constant α(b), impedance matching Z(c) and Eddy current loss C_o_(d).
