# Supplementary material for: Advanced N-Graphene-Based Electromagnetic Shields via Microwave Plasma Induced Synthesis
Source: Nanomaterials (Basel). 2026 Oct 25;16(21):1358. doi: 10.3390/nano16211358 (PMC13765024; doi:10.3390/nano16211358)
Supplement: Supplementary file 1 [file nanomaterials-16-01358-s001.zip › nanomaterials-4581319-supplementary.pdf]

Supporting Information

Advanced N-Graphene-Based Electromagnetic Shields via Microwave Plasma Induced Synthesis

*Elena Tatarova *, Neli Bundaleska *, Edgar Felizardo, Ana Dias, Antonio Silva, Miroslav Abrashev, Jivko Kissovski, Janez Zavašnik, Uroš Cvelbar, Nenad Bundaleski, Orlando M.N.D. Teodoro, Amelia Almeida, Milena Georgieva, Todor Karadimov and Bruno Gonçalves*

Supplementary Text

A schematic representation of the plasma-based synthesis process is shown in Figure 1S. A waveguide-surfatron based setup [1] is used to create a surface wave (SW) induced microwave plasma at atmospheric pressure conditions. The SW sustained discharge is produced by the field of a travelling wave (represented by the red lines in Figure 1S) that simultaneously propagates and creates its own propagation structure, sustaining the plasma along an extended active zone outside the wave launcher. In this way, large microwave power-densities can be injected into the processing area and high densities of relevant active species can be achieved. The discharge takes place inside a quartz tube with expanding radius, inserted perpendicularly to the waveguide wider wall and directed downstream. The design permits controlling the thermodynamic conditions (gas velocity, thermal fluxes, residence time, etc.) in the plasma reactor. The residence time can be controlled via the gas flow of background Ar gas, under vortex conditions.

The plasma reactor processes two distinguishable zones: a "hot" zone, sustained by surface waves and an afterglow "mild" plasma zone (Figure 1S). In the former, carbon/nitrogen precursors are introduced alongside a background flow of argon (Ar) under laminar or vortex conditions. Electrons absorb the wave power and consequently transfer it via elastic and non-elastic collisions to heavy particles and gas temperatures up to 4000 K can be achieved. Consequently, the injected carbonaceous molecules are decomposed into the fundamental components of carbon nanostructures, namely carbon atoms and C_2 radicals. Subsequently, the newly formed carbon atoms and C_2 molecules, are transported by the Ar gas flow towards the "mild" afterglow zone, characterized by lower gas temperatures ranging from 2000 K to 500 K.

Upon passing through a designated "vaporization surface," an isothermal plasma surface with a temperature of approximately 2500 K (Figure S1), gas-phase carbon atoms and C_2 molecules undergo a transformation into solid nuclei. This transition occurs within the larger volume of the "mild" zone, where kinetically driven processes of assembly and growth of free-standing flowing carbon nanostructures take place [2].
It must be noted that the synthesis of selected nanostructures with specific structural qualities is the result of a synergistic fine-tuning of the "hot" plasma environment and thermodynamic conditions in the "mild" zone. The set of operational parameters, including background gas and precursor partial fluxes, are meticulously adjusted to meet the prerequisites, i.e., to promote the self-assembly of planar sp^2 structures in the "assembly" zone of the plasma reactor. In essence, the engineered interplay of kinetic and thermodynamic factors allows for precise control over the nucleation and growth processes, paving the way for the tailored selective synthesis of graphene nanosheets.
Furthermore, to achieve a controlled *in situ* N-doping, acetonitrile injected from top in the "hot" plasma zone, was used both as a carbon and nitrogen precursor.
In order to synthesize nanocomposites, simultaneously with the generation of N-graphene sheets, a controlled flow of micron-sized particles Fe_2O_3, MnO_2 and TiO_2 nanoparticles is injected upstream at a specific position in the "mild" plasma zone. The particles rapidly heat up to the temperature of the background gas, which is around 2000 K in this zone. Due to the plasma's thermal and chemical reactor functions, the micron-sized particles undergo plasma-enabled size reduction and are converted into nanoparticles. Besides, due to the chemical reactions with radicals and molecules (H_2, C, CN, HCN, O_2 etc.) different chemical phases are created, and the nanoparticles are attached and/or anchored to the growing N-graphene sheets. Hundreds of milligrams of ready-to-use free-standing N-graphene sheets and graphene-based hybrids can be fabricated in minutes. In order to preserve graphene/N-graphene properties, protocols for synthesis of hybrid structures have been established targeting the maximization of the 2D-to-G ratio in the Raman spectra of the produce samples.
The following synthesis conditions were applied to synthesize NG-F-T hybrid: Q_{Ar} = 7.4 Slm; $Q_{Ar\ through\ C_2H_3N}$ = 150 sccm; $Q_{Ar\ through\ FexOy/Tio2}$ = 200 sccm; Z_{inj} = 23 cm; P = 5.4 kW. For NG-F-M hybrid: Q_{Ar} = 7.4 Slm; $Q_{Ar\ through\ C_2H_3N}$ = 150 sccm; $Q_{Ar\ through\ FexOy/MnO2}$ = 200 sccm; Z_{inj} = 23 cm; P = 5.4 kW (Figure S1).

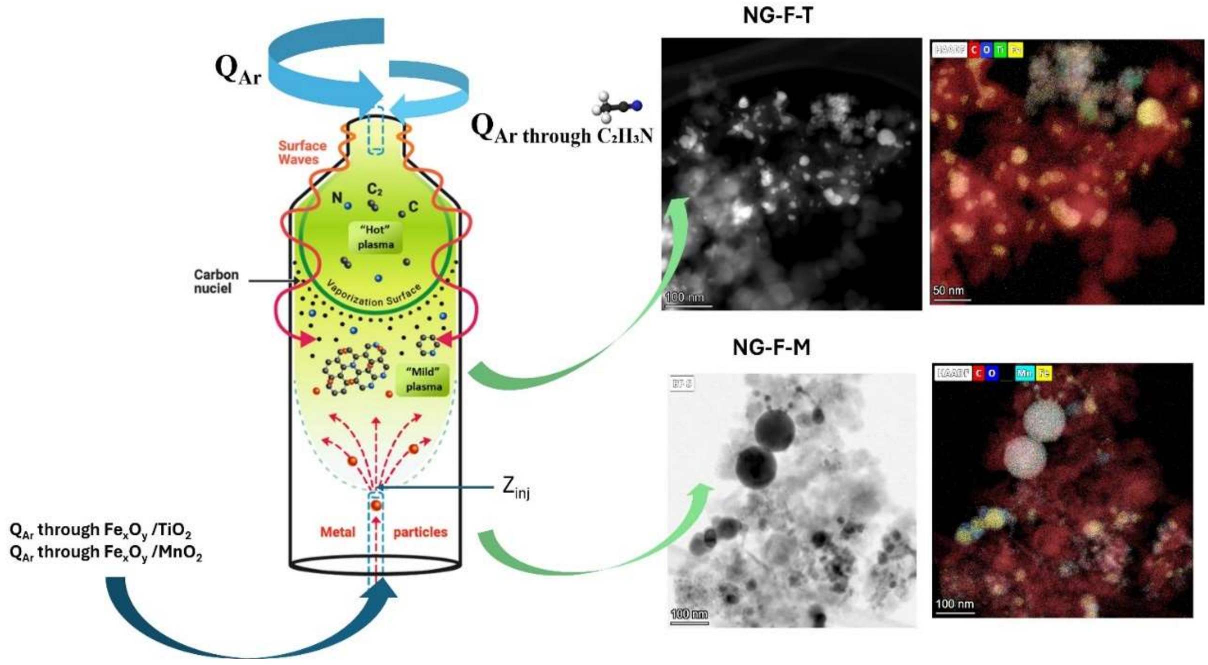

Figure S1. Schematic representation of the plasma reactor for free-standing N-graphene nanosheets and N-graphene hybrids synthesis. High-angle-annular dark-field (HAADF) STEM image of NG-F-T and NG-FM hybrids and EDS images.

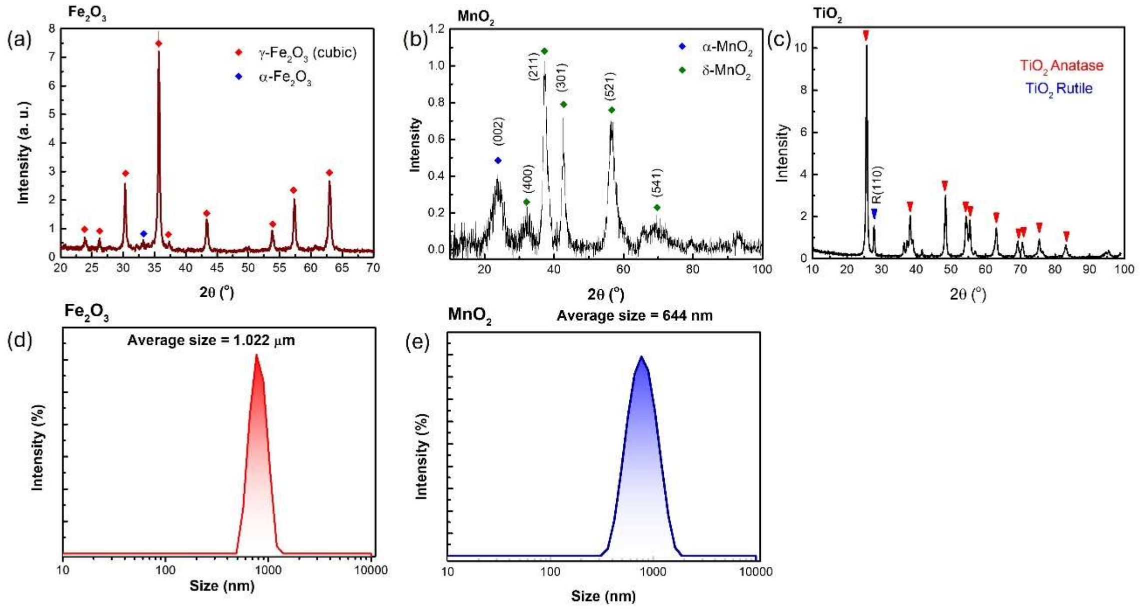

Figure S2. (a,b,c) XRD pattern of Fe_2O_3 MnO_2 and TiO_2 precursors, respectively; (d,e) DLS of Fe_2O_3 and MnO_2 precursor particles.

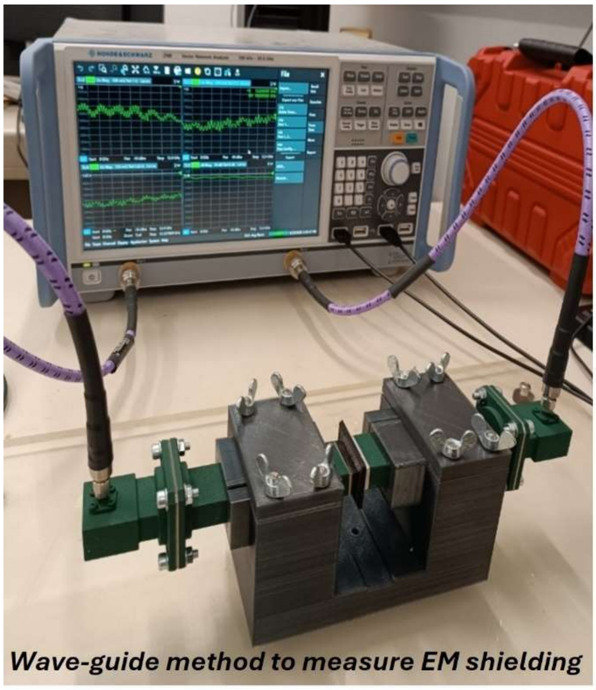

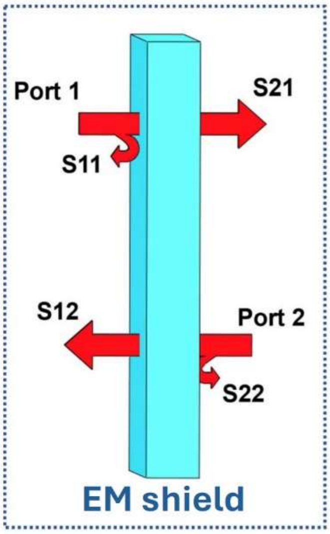

Figure S3. Experimental setup for measurements of scattering S-parameters.

The raw complex S-parameter data [3] were stored in Touchstone format $(.s2p)$ and the Power coefficients were extracted, from the gated S-parameters [4,5]. Energy balance $(R + T + A = 1)$ was verified across the frequency band as a consistency check.

Regarding measurements of S-parameters in K, Q, V, – bands the data were processed in MATLAB, using a custom algorithm incorporating two corrections to address artefacts inherent to bandpass VNA sweeps:

a) Band-edge artefact suppression. Prior to any spectral-to-temporal transformation, a Tukey (tapered cosine) window with shape parameter $\alpha = 0.20$ was applied to the complex S-parameter spectra [6]. This window tapers the outermost 5% of the band at each edge smoothly to zero while preserving unity weighting across the inner 90% of the bandwidth, thereby suppressing Gibbs ringing that would otherwise appear as spurious oscillations near the band edges after the inverse Fourier transform. After gating and back-transformation, the Tukey window was deconvolved by dividing the frequency-domain result by the window function within the flat region; the tapered edge points were excluded from further analysis.

b) Bandpass IFFT correction. Because the measurement sweep begins at a non-zero start frequency f_0, a direct application of the inverse fast Fourier transform (IFFT) to the measured $S(f)$ would incorrectly place the impulse response near $t = 0$, regardless of the true electrical delay of the fixture [7,8]. To recover the physically correct time-of-flight, the windowed $S(f)$ spectrum was first embedded into a full baseband frequency grid spanning $[0, f_{max}]$ with

frequency resolution Δf equal to the measurement step, yielding a zero-padded baseband representation. This grid was then further zero-padded by a factor of 8 ($NFFT\_FACTOR = 8$) to improve temporal interpolation, and a two-sided (Hermitian-symmetric) IFFT was applied to obtain a real-valued impulse response $h(t)$ [9].

For the Time-domain gating a Kaiser window [9,10] (shape parameter $\beta = 3$) was applied to $h(t)$ within a gate interval selected to bracket the primary sample response, set here to $[0.8, 4.8]$ ns while rejecting multipath reflections and fixture contributions outside this interval [7,8]. The gated impulse response $h_g(t)$ was then transformed back to the frequency domain via FFT, and the result was interpolated onto the original measurement frequency grid to yield the gated complex S-parameters.

Electromagnetic Parameters of Nanocomposites

NG-F-T-Epoxy nanocomposite

To demonstrate the synergy between different dielectric and magnetic loss mechanisms, the main electromagnetic parameters of nanocomposites under consideration have been deduced in Ku band (12-18 GHz) frequency range as an example. The Nicolson-Ross-Weir method has been used to extract dielectric permittivity and magnetic permeability from the measured scattering parameters [11].

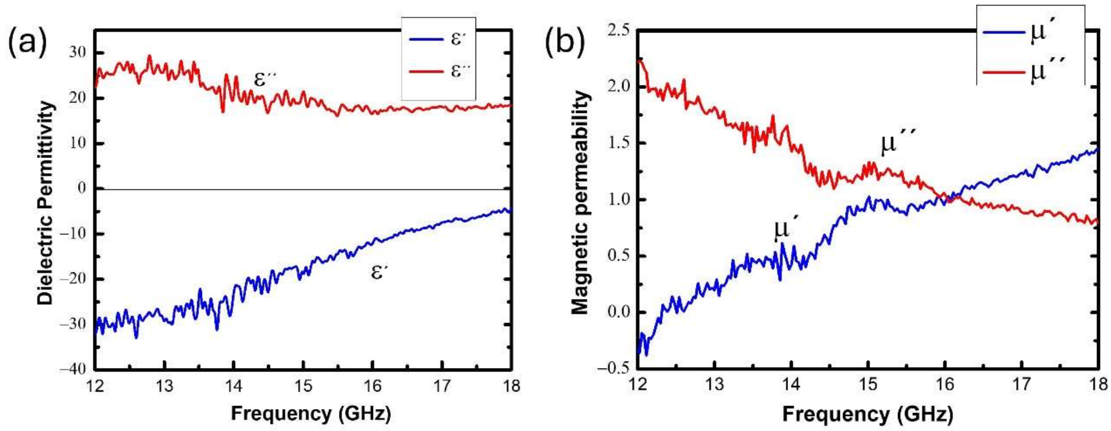

Figure S4. Real and imaginary parts of dielectric permittivity and magnetic permeability of NG-F-T_Epoxy nanocomposite.

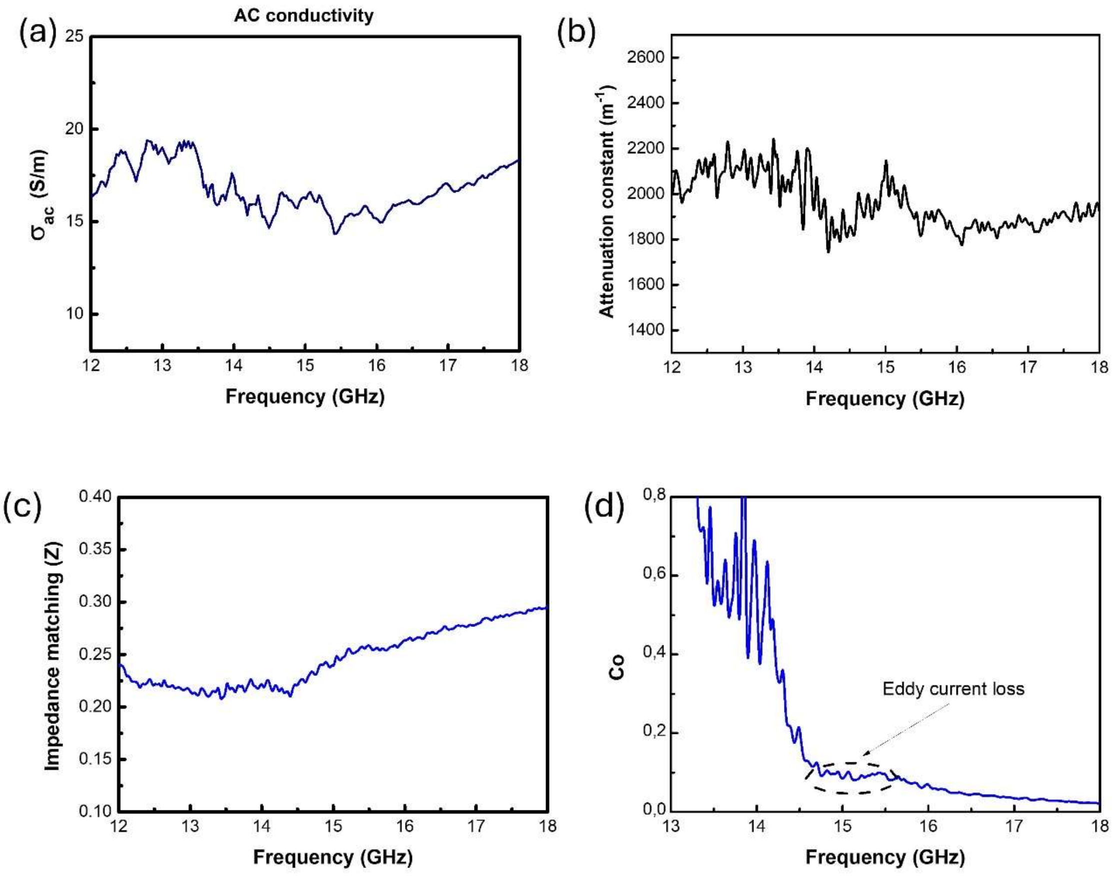

Figure S5. Electrical conductivity σ_{ac} (a), attenuation constant α (b), impedance matching Z (c) and Eddy current loss C_o(d).

Figure S4 (a,b) depicts real and imaginary parts of dielectric permittivity and magnetic permeability. The real part $\varepsilon'$of permittivity signifies the storage ability and is associated with the polarisation of the material. This includes dipolar polarisation due to nitrogen bonded groups, residual bonds and defects, and interfacial polarization arising between nanoparticles and graphene sheets under EM waves. Besides, the strong dielectric loss may benefit also from the surface plasmon resonances induced by N-graphene nano-islands ans metalic nanoparticles. For example, the N-graphene islands as plasmonic nanostructures exhibit collective oscillations, resulting in localized surface plasmon resonances under alternating EM fields. Surface plasmon resonances results in negative dielectric permittivity (12 - 18 GHz) as seen form (Fig. S4a) consistent with our previous studies [12].

The imaginary part $\varepsilon''$of permittivity refers to the dissipation ability of electric energy. Imaginary part of permittivity is higher than the real one resulting in higher absorption as compared with reflected power.

The calculated AC conductivity and the attenuation constant [13,14]

$$\varepsilon'' = \frac{\sigma_{ac}}{2\pi f \varepsilon_0}$$

$$\alpha = \frac{\sqrt{2}\pi f}{c}\sqrt{(\mu''\varepsilon'' - \mu'\varepsilon') + \sqrt{(\mu''\varepsilon'' - \mu'\varepsilon')^2 + (\varepsilon'\mu'' + \varepsilon''\mu')^2}}$$

are shown in Figure S5 (a,b).

The impedance matching controls how much EM wave can propagate inside the shielding material. [13,14]

$$Z = \sqrt{\mu_r/\varepsilon_r} = \sqrt{\sqrt{(\mu'^2 + \mu''^2)}/\sqrt{(\varepsilon'^2 + \varepsilon''^2)}}$$

In general, the EM wave absorption level of the shielding material is determined by impedance matching. For a good matching the value of impedance matching should be close to 1. As seen from Figure S5c the calculated impedance matching values are nearly constant ~0.25 and differs from one. As a result, high values of reflected power (>40%) are observed in the considered frequency range (Ku band) (Fig. 5b, see main article).

The real art of magnetic permeability μ' refers to the ability to storage of magnetic energy, while the imaginary μ'' part refers to the ability to dissipate the magnetic energy. Magnetic loss is influenced by multiple mechanisms including hysteresis loss, natural resonances, exchange resonances and eddy current loss. As seen from Figure 1b, the μ' values increase from 0 to one, while μ' decreases from 2 to nearly 1 in 12-18 GHz frequency range. Around 16 GHz a minimum of dissipated power is observed (Fig. 5b, see main article).

The eddy current loss can be evaluated using the following formula [13,14]

$$C_o = \mu''/(\mu'^2 f)$$

When the magnetic loss is solely caused by eddy current loss, then Co will be a constant. As seen from Figure S5d, Co changes significantly in the frequency range 12-18 GHz indicating week eddy current loss contribution considering NG-F-T-Epoxy nanocomposite. Such small contributions in the 14.5 - 15.5 GHz frequency range can be noted.

NG-F-M-Epoxy nanocomposite

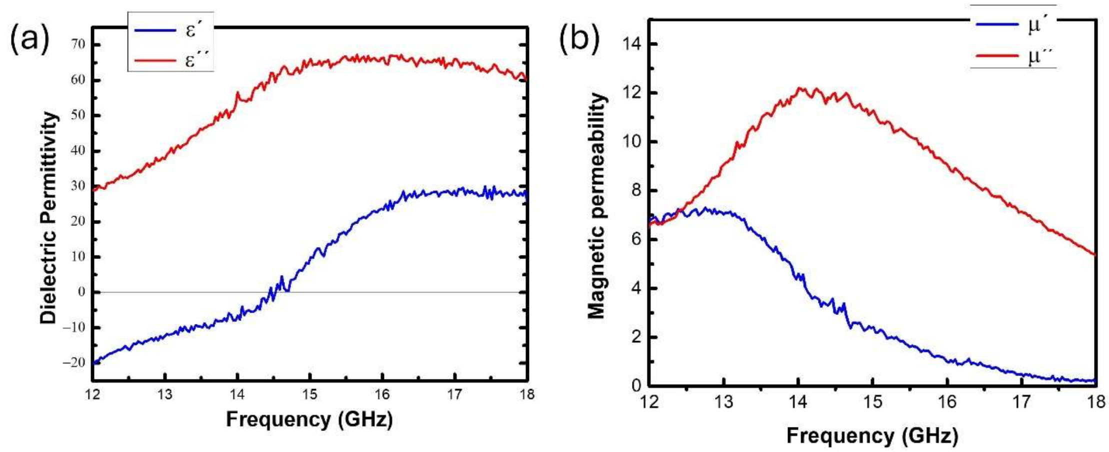

Figure S6. Real and imaginary parts of dielectric permittivity and magnetic permeability of NG-F-M-Epoxy nanocomposite.

Figure S6 (a,b) depicts real and imaginary parts of dielectric permittivity and magnetic permeability for NG-F-M-Epoxy nanocomposite. The real part ε´of permittivity changes from -20 to nearly 30 as frequency increased from 12 to 18 GHz. The presence of negative part in 12 - 14.5 GHz frequency rage could be a contribution of surface plasmon resonances relaxation to dielectric losses as shown in a previous study [12]. The relaxation of resonances results in conversion of electrical energy into thermal energy. Imaginary part ε´´ is higher than the real part ε´´. Consequently, a significant absorption (> 90%) of incident microwave power within the frequency range considered is observed (Fig. 6 a, see main article).

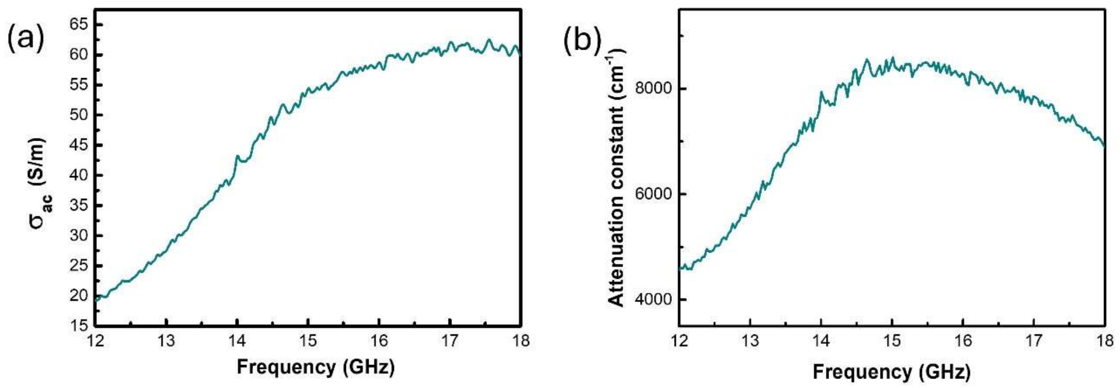

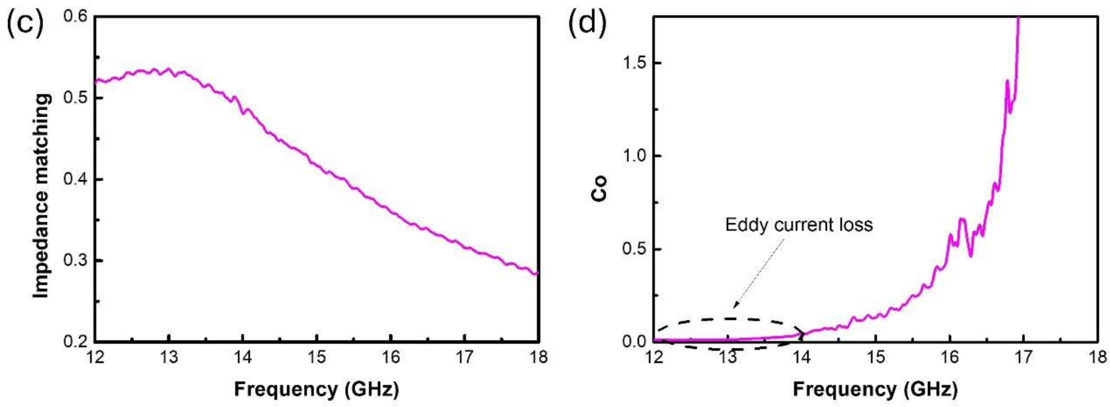

Figure S7. Electrical conductivity σ_{ac} (a), attenuation constant α(b), impedance matching Z (c) and Eddy current loss C_o(d).

AC conductivity and attenuation constant demonstrate (Fig. S7a,b) significantly higher values when compared with the corresponding parameters of NG-F-T-Epoxy nanocomposite, thus indicating high microwave dissipation through strong absorption. Impedance matching varies between approximately 0.5 to 0.3 in the 12 - 18 GHz frequency range (Fig. S7c) and correspondingly week reflected power (< 20%) is measured in the Ku band.

The variation of Co parameters with frequency (Fig. S7d) demonstrates synergystic contribution of natural and exchange resonances to magnetic losses in 14-18 GHz range while eddy current loss contribution could be more significant in 12-14 GHz range where Co is nearly constant.

References

1. Moisan, M.; Zakrzewski, Z. Plasma sources based on the propagation of electromagnetic surface waves. *J. Phys. D Appl. Phys.* **1991,** *24*, 1025.

2. Dias A. et al. Plasma-enabled multifunctional platform for gram-scale production of graphene and derivatives. *Applied Materials Today* **2024,** *36*, 102056.

3. IBIS Open Forum, "Touchstone® file format specification, version 2.1," IBIS Open Forum, Jan. 2024. [Online]. Available: https://ibis.org/touchstone_ver2.1/touchstone_ver2_1.pdf.

4. Pozar, D. M. *Microwave Engineering*, 4th ed. Hoboken, NJ, USA: Wiley, 2012.

5. Steer, M.B. Fundamentals of Microwave and RF Design. (3rd Edition), NC State University, 2019. doi: https//doi.org/10.5149/9781469656892.

6. Harris, F. J. On the use of windows for harmonic analysis with the discrete Fourier transform. *Proc. IEEE* **1978,** *66(1)*, 51–83. doi: 10.1109/PROC.1978.10837.

7. https://www.rohde-schwarz.com/products/test-and-measurement/vnas/rs-znb-vector-network-analyzer_63493-11648.html.

8. Anritsu Co., "Time domain measurements using vector network analyzers," Anritsu Co., Morgan Hill, CA, USA, Appl. Note. [Online]. Available: https://www.microwavejournal.com/ext/resources/BGDownload/d/3/Anritsu_Time.pdf.

9. Oppenheim, A. V.; Schafer, R. W.; Buck, J. R. *Discrete-Time Signal Processing*, 2nd ed. Upper Saddle River, NJ, USA: Prentice Hall, 1999.

10. Kaiser, J. F. Digital filters in *System Analysis by Digital Computer*, F. F. Kuo and J. F. Kaiser, Eds. New York, NY, USA: Wiley, (1966): 218-285.

11. Luukkonen, O.; Maslovski, S. I.; Tretyakov, S. A. A Stepwise Nicolson–Ross–Weir-Based Material Parameter Extraction Method, December 2011, *IEEE Antennas and Wireless Propagation Letters* **2011,** *10,*1295-1298. DOI:10.1109/LAWP.2011.2175897.

12. Tatarova, E.; Dias, A.; Dankov P. et. al. Plasma-Driven Tuning of Dielectric Permittivity in Graphene. *Small* **2024,** *20*, 2303421. https://doi.org/10.1002/smll.202303421.

13. Anju, et al. High-Performance, Lightweight, and Flexible Thermoplastic Polyurethane Nanocomposites with Zn^{2+}-Substituted $CoFe_2O_4$ Nanoparticles and Reduced Graphene Oxide as Shielding Materials against Electromagnetic Pollution. *ASC Omega* **2021,** 6, 28098. https://doi.org/10.1021/acsomega.1c04192.

14. Li, S.; Sun, Y.; Zhang, K.; Jiang, X.; Yu, H. In Situ Fabrication of Heterogeneous Co/Nanoporous Carbon Nano-Islands for Excellent Electromagnetic Wave Absorption. *Small* 2024, *20*, 2306990. https://doi.org/10.1002/smll.202306990.
